# Oriental Hornet (*Vespa orientalis*) Larval Extracts Induce Antiproliferative, Antioxidant, Anti-Inflammatory, and Anti-Migratory Effects on MCF7 Cells

**DOI:** 10.3390/molecules26113303

**Published:** 2021-05-31

**Authors:** Amina M. G. Zedan, Mohamed I. Sakran, Omar Bahattab, Yousef M. Hawsawi, Osama Al-Amer, Atif A. A. Oyouni, Samah K. Nasr Eldeen, Mohammed A. El-Magd

**Affiliations:** 1 Biological and Environmental Sciences Department, Home Economic Faculty, Al Azhar University, Tanta 31732, Egypt; AminaZedan1948.el@Azhar.edu.eg; 2 Biochemistry Department, Faculty of Science, University of Tabuk, Tabuk 47512, Saudi Arabia; msakran@ut.edu.sa; 3 Biochemistry Division, Chemistry Department, Faculty of Science, Tanta University, Tanta 31512, Egypt; 4 Biology Department, Faculty of Science, University of Tabuk, Tabuk 47512, Saudi Arabia; Obahattab@ut.edu.sa; 5 Research Center, King Faisal Specialist Hospital and Research Center, MBC J04, Jeddah 21499, Saudi Arabia; hyousef@kfshrc.edu.sa; 6 College of Medicine, Al-Faisal University, Riyadh 11533, Saudi Arabia; 7 Department of Medical Laboratory Technology, Faculty of Applied Medical Sciences, University of Tabuk, Tabuk 47512, Saudi Arabia; oalamer@ut.edu.sa; 8 Genome and Biotechnology Unit, Faculty of Sciences, University of Tabuk, Tabuk 47512, Saudi Arabia; a.oyouni@ut.edu.sa; 9 Department of Biology, Faculty of Sciences, University of Tabuk, Tabuk 47512, Saudi Arabia; 10 Central Laboratories, Egyptian Ministry of Health, Tanta 31512, Egypt; sciencesamah@gmail.com; 11 Department of Anatomy, Faculty of Veterinary Medicine, Kafrelsheikh University, Kafrelsheikh 33516, Egypt

**Keywords:** *Vespa orientalis*, MCF7, apoptosis, antioxidant, anti-migration, anti-inflammatory

## Abstract

The use of insects as a feasible and useful natural product resource is a novel and promising option in alternative medicine. Several components from insects and their larvae have been found to inhibit molecular pathways in different stages of cancer. This study aimed to analyze the effect of aqueous and alcoholic extracts of *Vespa orientalis* larvae on breast cancer MCF7 cells and investigate the underlying mechanisms. Our results showed that individual treatment with 5% aqueous or alcoholic larval extract inhibited MCF7 proliferation but had no cytotoxic effect on normal Vero cells. The anticancer effect was mediated through (1) induction of apoptosis, as indicated by increased expression of apoptotic genes (*Bax*, caspase3, and *p53*) and decreased expression of the anti-apoptotic gene *Bcl2*; (2) suppression of intracellular reactive oxygen species; (3) elevation of antioxidant enzymes (CAT, SOD, and GPx) and upregulation of the antioxidant regulator *Nrf2* and its downstream target *HO-1*; (4) inhibition of migration as revealed by in vitro wound healing assay and downregulation of the migration-related gene *MMP9* and upregulation of the anti-migratory gene *TIMP1*; and (5) downregulation of inflammation-related genes (*NFκB* and *IL8*). The aqueous extract exhibited the best anticancer effect with higher antioxidant activities but lower anti-inflammatory properties than the alcoholic extract. HPLC analysis revealed the presence of several flavonoids and phenolic compounds with highest concentrations for resveratrol and naringenin in aqueous extract and rosmarinic acid in alcoholic extract. This is the first report to explain the intracellular pathway by which flavonoids and phenolic compounds-rich extracts of *Vespa orientalis* larvae could induce MCF7 cell viability loss through the initiation of apoptosis, activation of antioxidants, and inhibition of migration and inflammation. Therefore, these extracts could be used as adjuvants for anticancer drugs and as antioxidant and anti-inflammatory agents.

## 1. Introduction

Despite remarkable advances in chemotherapy, the numerous side effects and non-selective targeting of most of the currently used anticancer drugs restrict their therapeutic potential [1,2]. Thus, discovering novel anticancer agents with superior inhibitory effect and selective targeting to cancer cells is becoming an urgent need [3,4,5]. In recent years, targeted therapy has been searching for natural and safe medicines to avoid the side effects of anticancer drugs [6,7]. Extracts prepared from natural products and medicinal plants have notable anti-inflammatory, antioxidant and anticancer potential [3,4,5,6,7]. Similarly, many bioactive ingredients extracted from insects have demonstrated anticancer effects [8,9,10,11]. Insects and their larvae could be considered as a good source for many bioactive compounds; however, their role has not been fully investigated [12]. Previous studies have reported that some insect larvae, such as housefly larvae, possess anticancer, antioxidant, and antimicrobial properties [11,13,14,15,16]. Additionally, aqueous extracts and hydrolysates of some insects such as house cricket, grasshopper, and silk moth contain some beneficial bioactive peptides that have anti-inflammatory and antioxidant activities [17,18]. In addition to these beneficial medicinal effects, insects are part of the common diet of at least two billion people in the world, as they contain high amounts of proteins and micronutrients [19]. Consumption of antioxidant-rich nutrients helps in preventing oxidative stress-induced diseases and cancer [5,20,21,22,23].

Oriental hornets (*Vespa orientalis*) are insects that belong to the family Vespidae, which are distributed in the Middle East, Southwest Asia, Southern Europe, and Northeast Africa, and live in colonies [24]. The fertilized queens hibernate in the winter [25,26], lay their eggs during the fall [27], and the population of the colony peaks in the late summer and initial fall [28]. These insects attack bee colonies to get nectar and protein, causing notable loss to honey bee production [29,30]. Although their stings are highly painful to humans and can cause allergy, they are used for the treatment of cold and gastritis in some Indian tribes [26]. These tribes also consume various *Vespa sp*. due to their high protein content [31]. Moreover, the aqueous extract of lesser banded hornet (*V. affinis*) has been shown to exert antioxidant effects through activation of the antioxidant enzymes GST and CAT [32]. Venom extracted from *V. orientalis* showed a potent antimicrobial effect against a large variety of bacteria [33,34,35] and an anticancer effect [36,37,38]. One of the major drawbacks of using insect venoms as an anticancer agent is their cytotoxic and neurotoxic effects on normal cells [39,40]. Using insect larva and pupa could be a useful alternative. However, to date, there have been no reports investigating the antioxidant and anticancer effects of *V. orientalis* larval extracts.

Therefore, this study aimed to assess the anticancer effect of aqueous and alcoholic extracts of *V. orientalis* larvae against MCF7 cells and to study the underlying mechanisms through investigating apoptotic pathway, antioxidant status, migration, and inflammation.

## 2. Material and Methods 

### 2.1. Preparation of V. orientalis Larval Extracts

Nests of *V. orientalis* were collected in the fall of 2019 from different localities in the cities of Tanta and Kafrelsheikh, Egypt. The insects and nests were kept under controlled conditions with respect to temperature, relative humidity, and photoperiods in the Honey Bee Laboratory at Plant Protection Department, Faculty of Agriculture, Kafrelsheikh University. A standard rearing method was used to obtain the larvae required for the bioassay. The 3rd and 4th larvae instars were extracted using 5% water or ethyl alcohol as previously described [32,41]. In brief, the collected larvae were first washed in distilled water, and a mixture of 5 g larvae and 95 mL distilled water or pure ethyl alcohol was centrifuged (8000 rpm/20 min/4 °C) to eliminate undesirable debris. The supernatant was obtained and considered as 5% aqueous and alcoholic extracts and kept in the freezer until further use.

### 2.2. HPLC Analysis of Flavonoids and Phenolic Compounds

HPLC was used to analyze flavonoids and phenolic compounds in both aqueous and alcoholic extracts, as previously described [42]. The HPLC (Agilent1260 infinity HPLC Series, Agilent Technologies, Santa Clara, CA, USA) was equipped with a Quaternary pump and Kinetex 5 μm EVO C18 (100 mm × 4.6 mm) column (Phenomenex, Torrance, CA, USA) operated at 30 °C. The separation was achieved using a ternary linear elution gradient with HPLC grade water 0.2% H3P04 (*v/v*), methanol, and acetonitrile. The injected volume was 20 μL and the VWD detector was set at 284 nm.

### 2.3. DPPH Radical Scavenging Assay

A DPPH (2,2-diphenyl-1-picryl-hydrazil) radical scavenging assay was performed as previously described [43]. A methanolic solution of DPPH reagent (1 mL, 0.004%) was added to each larval extract (100 µL) dissolved in DMSO at different concentrations (20, 40, 80, 160, and 320 µg/mL). Following 1 h incubation in the dark, a yellow color developed, and the absorbance was recorded at 515 nm. Ascorbic acid was used as a positive control. The IC_50_ value was calculated using GraphPad prism. The experiment was repeated three times. The DPPH scavenging activity was calculated from this equation: DPPH scavenging (%) = (A0 − A1)/A0 × 100, where A0 is the control absorbance and A1 is the sample absorbance.

### 2.4. MTT Cytotoxicity Assay 

Human breast adenocarcinoma MCF7 cells and normal African green monkey kidney Vero cells were obtained from VACSERA, Cairo. MTT assay was performed as previously described [44]. Cells were seeded at a concentration of 1 × 10^4^ cells per well. DMEM medium, 10% FBS, 1% penicillin/streptomycin, and 2% L-glutamine (all supplemented from Gibco, Waltham, MA, USA) were added to each well, and cells were incubated at 37 °C, in a humidified atmosphere of 95% air and 5% CO_2_ for 24 h until 80–90% confluence. Serial dilutions (0, 12.5, 25, 50, 100, and 200 μg/mL) of each *V. orientalis* larval extract were applied to cells. Following incubation for 1 day, 5 mg/mL of 3-(4,5-Dimethylthiazol-2-yl)-2,5-diphenyltetr-azolium bromide (MTT, Invitrogen, Waltham, MA, USA) was added, and cells were incubated for 4 h; then, the medium was removed and substituted with 100 μL Dimethyl sulfoxide (DMSO, Sigma Aldrich, St. Louis, MO, USA). Finally, the absorbance was measured at 570 nm.

### 2.5. Experimental Design

The cells were allocated into three groups. The untreated MCF7 cells were considered control cells (Cnt), while in the other two groups, cells were treated with aqueous or alcoholic larval extracts with doses equal to their IC_50_. Treatments were applied in triplicate at 70–80% confluence. Cells were incubated in a CO_2_ incubator for 24 h at 37 °C and 95% humidity.

### 2.6. Estimation of Intracellular Reactive Oxygen Species 

Intracellular reactive oxygen species (ROS) were determined using a fluorescent probe, 2′,7′-dichlorofluorescein diacetate (DCFDA), as previously described [45]. MCF7 cells were treated with aqueous and alcoholic larval extracts with doses equal to their IC_50_ for 2 h followed by 25 μM H_2_O_2_ for 2 h. After PBS washing, DCFDA (5 μM) was added, and the cells were incubated (37 °C/30 min) in the dark. Following washing in PBS, the fluorescence intensity of DCFDA was measured in a fluorescence microplate reader. The intracellular ROS levels were calculated as a % of the control.

### 2.7. Detection of Antioxidant Enzymes Activities

The activities of superoxide dismutase activity (SOD), catalase (CAT), glutathione peroxidase (GPx) in MCF7 cells were assayed by colorimetric methods using commercially available kits and following the manufacturer’s protocol (Biodiagnostics, Cairo, Egypt). The antioxidant activities were presented as percentages of the control.

### 2.8. In Vitro Scratch (Wound Healing) Assay

MCF7 cells were grown at a concentration of 2.5 × 10^5^ cells/mL in 6-well plates in DMEM until 75% confluence. A scratch was produced in the center of each well and fresh media containing each extract at a concentration equal to IC_50_ were added. Images were taken at two time intervals (0 h and 24 h), and MCF7 migration was calculated as previously reported [46].

### 2.9. Gene Expression Analysis by qPCR 

RNA samples were extracted from all cells using a procedure detailed previously [6]. Nanodrop and 1% gel electrophoresis were used to determine RNA concentration and integrity, respectively. Following reverse transcription using RevertAid H Minus Reverse Transcriptase, the expression of *Bax*, *Bcl2*, caspase 3, *p53*, *NrF2*, *HO-1*, *MMP9*, *TIMP1*, *NFκB*, and *IL8* genes was determined using 2X SYBR Green Master Mix and specific primers (Table 1). All kits used in qPCR were purchased from Thermo Scientific, Waltham, MA, USA. The qPCR mixture (25 μL) contained 2 μL cDNA, 1 μL from each primer and 12.5 μL of Maxima SYBR Green Master Mix. The thermal cycling included 10 min at 95 °C followed by 45 cycles of (95 °C for 15 s, 60 °C for 30 s and 72 for 30 s). The relative expression of target genes was normalized with the housekeeping gene *GAPDH* and calculated using the 2^−ΔΔCt^ method [47].

### 2.10. Statistical Analysis

All data were expressed as mean ± standard error of the mean (SEM) of replicates from independent experiments. The difference between the groups was evaluated by one-way analysis of variance and Tukey’s Honest Significant Difference test using GraphPad prism, 7.0 software. Values were considered statistically significant when *p* < 0.05.

## 3. Results

### 3.1. Larval Extract Had Numerous Flavonoids and Phenolic Compounds

**Figure 1:**
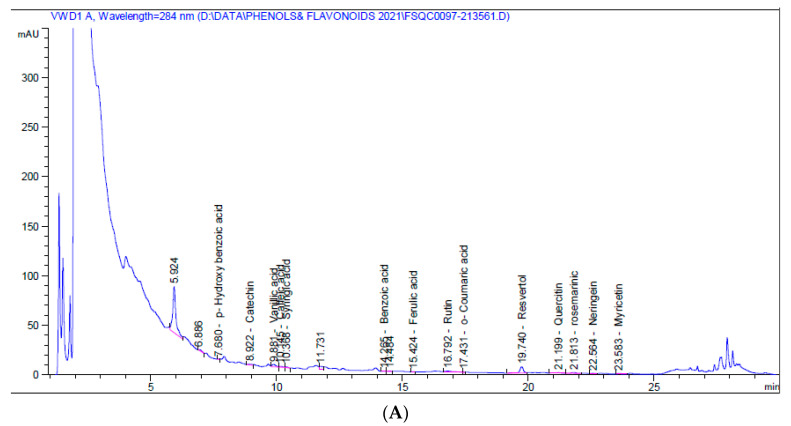

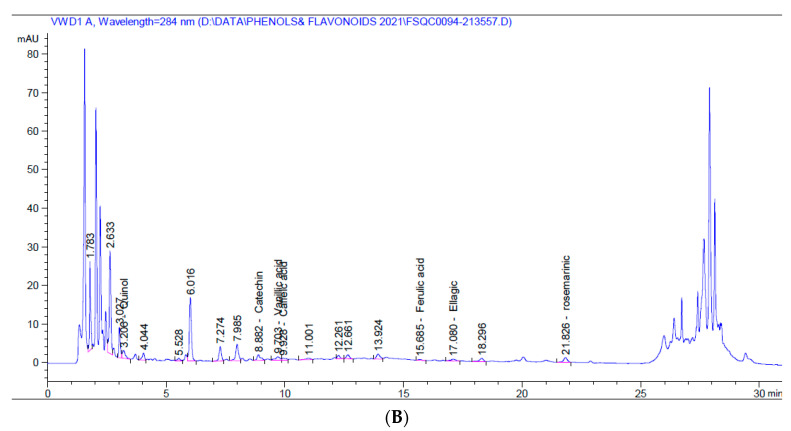
HPLC chromatograms of 5% aqueous (**A**) and alcoholic (**B**) larval extracts.

HPLC chromatograms for the 5% aqueous larval extract revealed the presence of numerous flavonoids (catechin, chlorogenic, rutin, quercetin, naringenin, myricetin) and phenolic compounds (vanillic acid, caffeic acid, syringic acid, ferulic acid, o-coumaric acid, resveratrol, and rosmarinic acid), as well as benzoic acid and p-hydroxybenzoic acid (a phenolic derivative of benzoic acid), p-coumaric acid, benzoic acid, and cinnamic acid. Resveratrol was the major phenolic compound (1.580 mg/kg at 19.74 min), while naringenin was the major flavonoid (0.819 mg/kg at 22.56 min) (Table 2, Figure 1A). 

On the other hand, the 5% alcoholic larval extract contained only catechin flavonoid and fewer phenolic compounds (vanillic acid, caffeic acid, ferulic acid, ellagic acid, and rosmarinic acid). However, the total phenolic compounds and flavonoids (45.460 mg/kg) were higher than those of the 5% aqueous larval extract (4.452 mg/kg). The major phenolic compound obtained from the alcoholic larval extract was rosmarinic acid (34.031 mg/kg at 21.83 min), which was responsible for this elevation (Table 2, Figure 1B). Looking at the HPLC chromatograms displayed in Figure 1, other unknown higher peaks can be observed that were not identified due to lack of available standards.

### 3.2. Larval Extract Had Potent Antioxidant Activities 

**Figure 2:**
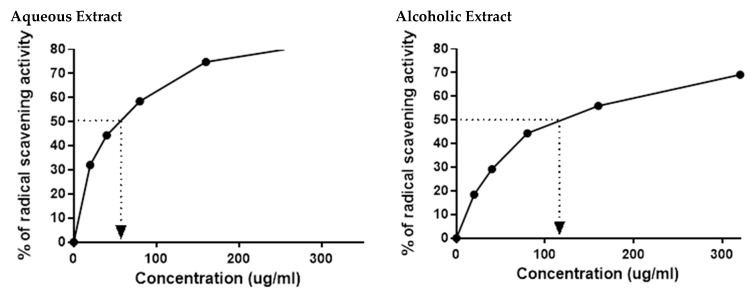
DPPH radical scavenging activity of 5% aqueous and alcoholic larval extracts shows their IC_50_ values (52.67 ± 2.38 and 123.50 ± 4.62 µg/mL, respectively). Values are mean ± SEM. Samples were run in triplicate in three independent experiments, *n* = 5.

The HPLC results indicated the presence of numerous flavonoids and phenolic compounds, which all had antioxidant properties. This prompted us to confirm the in vitro antioxidant activities of the two larval extracts using DPPH assay. As expected, 5% aqueous and alcoholic larval extracts had potent free radical scavenging activity, with IC_50_ values of 52.67 ± 2.38 and 123.50 ± 4.62 µg/mL, respectively, relative to the standard ascorbic acid (45.33 µg/mL) (Figure 2). This suggests that the 5% aqueous larval extract had better free radical scavenging activity than the 5% alcoholic larval extract. 

### 3.3. Larval Extracts Inhibited MCF7 Viability

**Figure 3:**
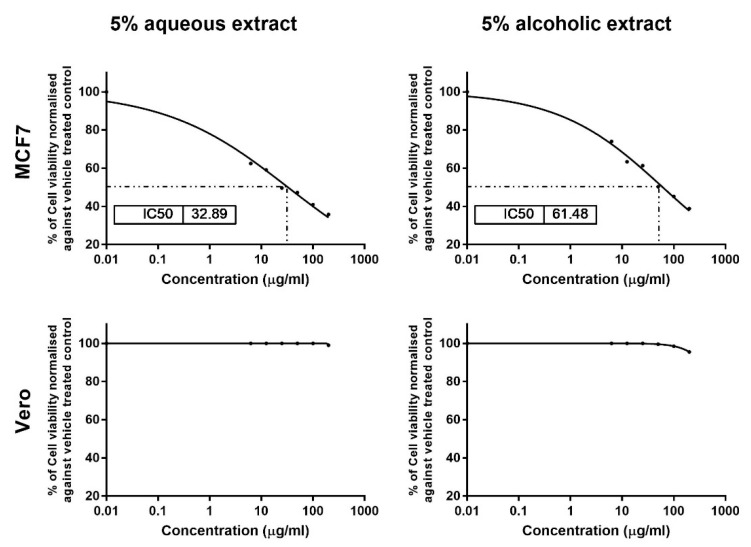
Cytotoxicity analysis of the 5% larval extracts using the MTT assay. Dose–response curves show the effect of the two extracts on MCF7 and Vero viability and the obtained IC_50_ after 24 h. Data were normalized to untreated control cells and expressed as the mean % viability ± SEM. Samples on MCF7 and Vero were run in triplicate in three independent experiments, *n* = 5.

The results of MTT assay showed dose-dependent cytotoxic effects for 5% aqueous and alcoholic larval extracts on MCF7, with IC_50_ values of 32.89 ± 1.84 and 61.48 ± 2.70 µg/mL, respectively, as compared to control (untreated) cells (Figure 3). This suggests that the aqueous larval extract had a more potent anticancer effect than the alcoholic larval extract. However, the aqueous extract showed no cytotoxic effect on normal Vero cells up to a concentration of 100 µg/mL, and only 1% cell viability inhibition was noticed at a concentration of 200 µg/mL. The alcoholic extract also showed minimal inhibition of 0.50%, 1.5% and 4.5% at concentrations of 50, 100, and 200 µg/mL, respectively (Figure 3).

### 3.4. Larval Extracts Modulated the Expression of Apoptosis-Related Genes 

**Figure 4:**
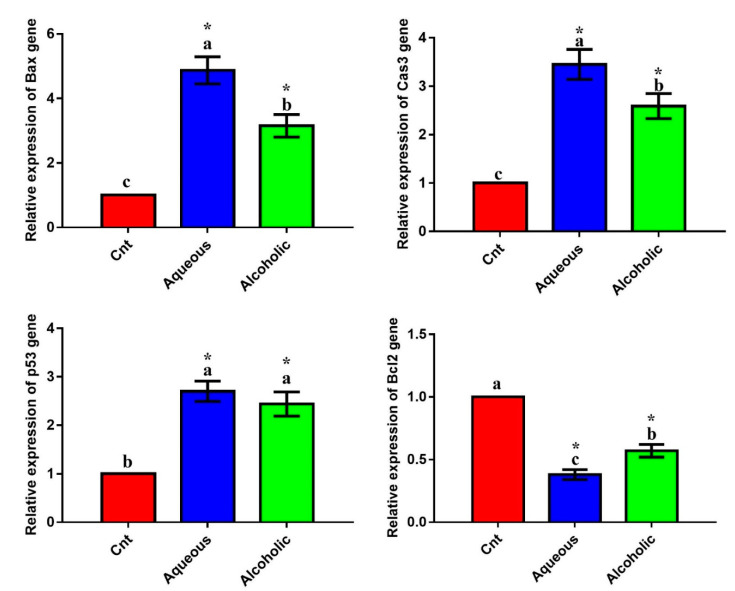
Expression of *Bax*, caspase3 (*Cas3*), *p53*, and *Bcl2* genes in MCF7 cells following treatment with 5% aqueous and alcoholic larval extracts as detected by qPCR. Data were normalized to the housekeeping gene (*GAPDH*) and are expressed as the mean fold change ± SEM. Samples were run in triplicate in three independent experiments, *n* = 5. Groups with different letters are significantly different at *p* < 0.05. * indicates high significance with respect to control (*p* < 0.001).

The qPCR was applied to monitor the effect of treatments on the expression of the apoptotic genes (*Bax*, caspase3, and *p53*) and the anti-apoptotic gene (*Bcl2*). Treatment with each extract significantly upregulated the expression of the apoptotic genes and significantly downregulated the expression of *Bcl2* compared to the control cells (Figure 4). The 5% aqueous extract showed higher expression of *Bax* and caspase3 and lower expression of *Bcl2* than the 5% alcoholic extract. These results suggest that the cytotoxic effect of the two extracts on MCF7 cells could be triggered by apoptosis. On the other hand, no significant difference was observed in any of these apoptotic or anti-apoptotic genes among the three groups in Vero cells (Figure S1). These results imply that the two larval extracts would not induce apoptosis in normal Vero cells. 

### 3.5. Larval Extracts Inhibited Intracellular ROS and Induced Antioxidant Status

**Figure 5:**
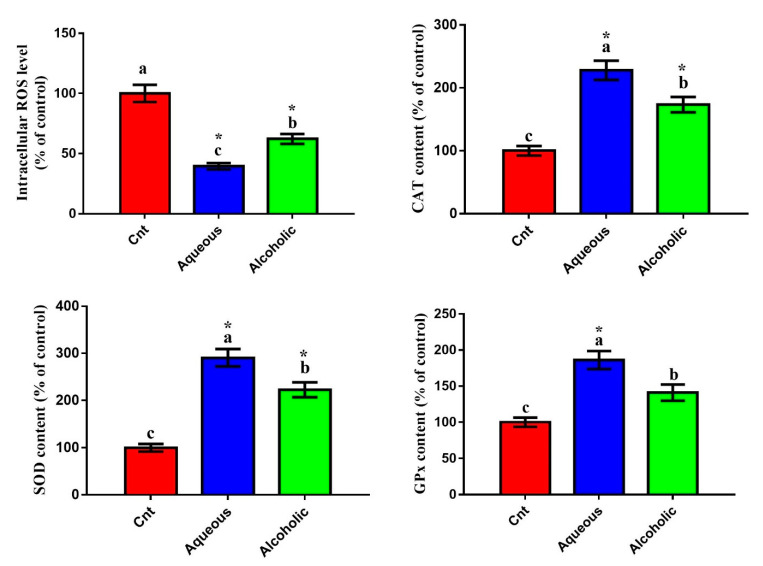
Effect of 5% aqueous and alcoholic larval extracts on intracellular ROS and activities of antioxidant enzymes (CAT, SOD, and GPx) in MCF7 cells. Data are expressed as % of the control ± SEM. Samples were run in triplicate in three independent experiments, *n* = 5. Groups with different letters are significantly different at *p* < 0.05. * indicates high significance with respect to the control (*p* < 0.01).

Given that *V. orientalis* larval extracts contained numerous flavonoids and phenolic compounds and had potent total antioxidant activities, we postulated that these extracts could trigger cancer cell death through decreasing oxidative stress and increasing antioxidant enzyme activities. To assess this hypothesis, we evaluated the effect of these extracts on the intracellular ROS and activities of antioxidant enzymes. Increased intracellular ROS is a notable indicator of cellular oxidative stress. As was expected, cells treated with each larval extract showed significantly lower intracellular ROS and significantly higher levels of CAT, SOD, and GPx than control cells (Figure 5). Again, the 5% aqueous extract exhibited lower intracellular ROS and higher levels of CAT, SOD, and GPx than the 5% alcoholic extract. 

**Figure 6:**
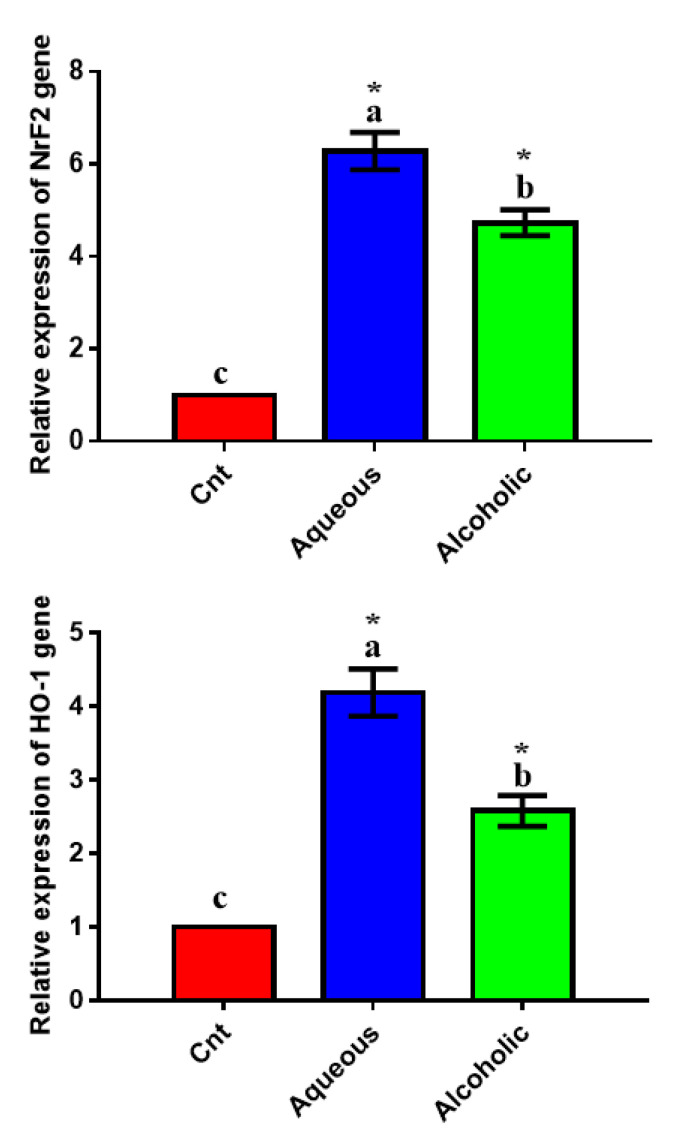
Expression of *NrF2* and *HO-1* genes in MCF7 cells following treatment with 5% aqueous and alcoholic larval extracts as detected by qPCR. Data were normalized to the housekeeping gene (*GAPDH*) and are expressed as the mean fold change ± SEM. Samples were run in triplicate in three independent experiments, *n* = 5. Groups with different letters are significantly different at *p* < 0.05. * indicates high significance with respect to control (*p* < 0.001).

To confirm the antioxidant potential of the two extracts on a molecular basis, we studied their effect on the expression of the antioxidant-regulator *NrF2* and its downstream target *HO-1* using qPCR and found significant upregulation of the two genes in MCF7 cells treated with each extract, with higher expression in 5% aqueous extract-treated cells than in control cells (Figure 6).

### 3.6. Larval Extracts Inhibited MCF7 Migration

**Figure 7:**
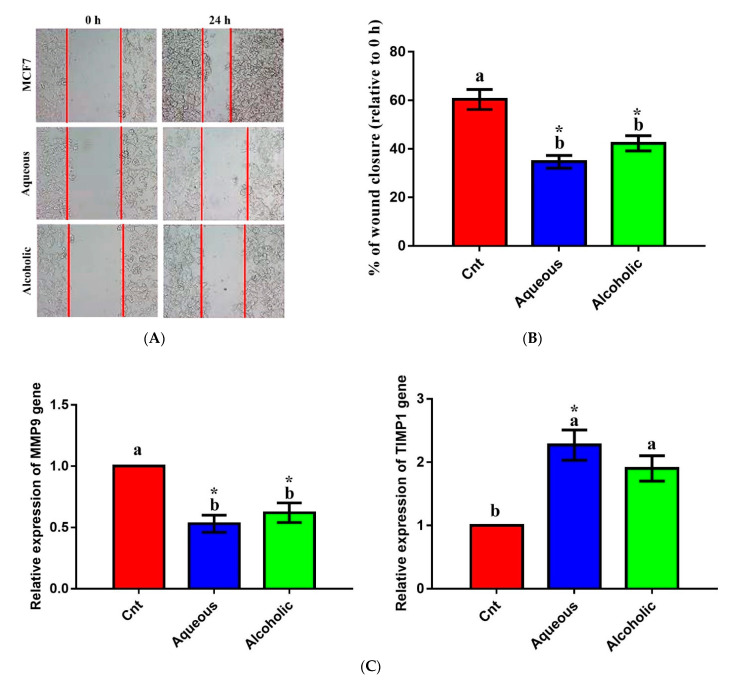
Anti-migratory effect of 5% aqueous and alcoholic larval extracts on MCF7 cells. (**A**) Wound-healing assay. (**B**) Percentage of wound closure of MCF7 cells. (**C**) Expression of *MMP9* and *TIMP1* genes in MCF7 cells. Data are expressed as the mean ± SEM. Samples were run in triplicate in three independent experiments, *n* = 5. Groups with different letters are significantly different at *p* < 0.05. * indicates high significance with respect to the control (*p* < 0.01).

The effect of larval extracts on MCF7 migration was evaluated using a scratch (wound-healing) assay. Treatment with each larval extract significantly inhibited MCF7 migration compared to the control (Figure 7). The rates of MCF7 migration after treatment with aqueous and alcoholic extracts were 34.68 ± 2.63% and 42.28 ± 3.14%, respectively, compared to the control cells (60.38 ± 4.12%). To verify this anti-migratory effect on a molecular level, qPCR was used to check changes in the relative expression of the migration-related *MMP9* and the anti-migratory *TIMP1* following treatment with each larval extract. The obtained results revealed significant downregulation of *MMP9* and upregulation of *TIMP1* in cells treated with each extract relative to control cells (Figure 7). No significant change in *MMP9* or *TIMP1* was noticed between the two extracts.

### 3.7. Larval Extracts Reduced the Expression of Inflammation-Related Genes 

**Figure 8:**
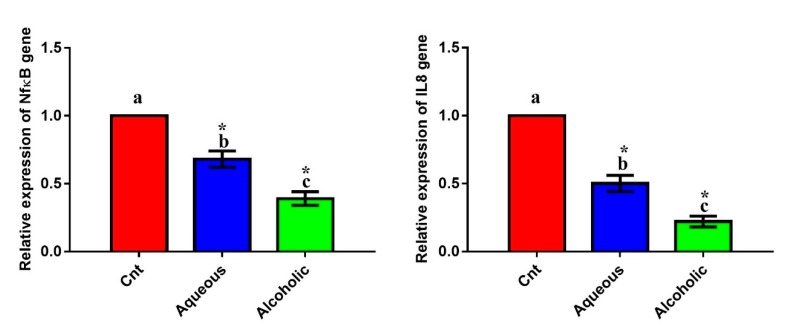
Expression of *NFκB* and *IL8* genes in MCF7 cells following treatment with 5% larval aqueous and alcoholic extracts. Data are expressed as the mean ± SEM. Samples were run in triplicate in three independent experiments, *n* = 5. Groups with different letters are significantly different at *p* < 0.05. * indicates high significance with respect to the control (*p* < 0.01).

The inflammatory genes *NFκB* and *IL8* exhibited a significant downregulation after the addition of each extract compared to the control cells (Figure 8). The most downregulated effect was encountered in cells treated with the 5% alcoholic extract. These findings indicate that these extracts have an anti-inflammatory effect against MCF7 cells, with the best effect being for the 5% alcoholic extract.

## 4. Discussion

There is growing interest in exploring the roles of the insects’ bioactive components, especially those with antioxidant and anticancer properties. Previous studies have only investigated the anticancer effect of *Vespa sp.* venom and its bioactive peptides [36,37,38]. However, the effect of the venom is not selective and can damage normal cells [39,40]. Aside from the venom, insect developmental stages (larvae and pupae) exhibit antioxidant and anticancer potential, with less toxicity for normal cells. Dutta et al. [32] reported an antioxidant effect for *V. affinis* aqueous pupal extract. Moreover, the larval hemolymph of *M. domestica* had antioxidant and cytotoxic properties against MCF7, but no cytotoxicity on normal Vero cells [11]. To the best of our knowledge, this is the first study to report that 5% *V. orientalis* larval extracts had an anticancer effect on MCF7 cells, and that this effect could be mediated by, at least in part, the induction of apoptosis, activation of antioxidants, and inhibition of migration and inflammation. 

Previous studies have demonstrated cytotoxic effects for *V. orientalis* venom and attributed this effect to a bioactive peptide called mastoparan, which causes membrane destabilization and subsequent cell lysis [48,49]. Mastoparan also activates G-protein, which subsequently initiates mitochondrial permeability and apoptosis [50]. However, little is known about the effect of *V. orientalis* larval extracts on cancer cells. In the present study, we studied this effect and reported a notable dose-dependent antiproliferative potential for *V. orientalis* larval extracts on MCF7 with better influence for the aqueous extract which showed lower IC_50_ (32.89 ± 1.84 µg/mL) than the alcoholic extract (61.48 ± 2.70 µg/mL). This indicates a more potent anticancer effect for the aqueous larval extract. Unlike most insect venoms, these extracts are less toxic on normal cells such as Vero cells, indicating a high safety margin. This is in agreement with the safe consumption of many *Vespa Sp.* by some tribes in India [31]. However, in vivo investigations on animal models of cancer are required to validate their biosafety and choose the optimal doses.

Similar to *V. orientalis* venom and its ingredient mastoparan, the cytotoxic effect of larval extracts was mediated through induction of apoptosis as indicated by significant elevation of the three apoptotic genes *Bax*, caspase 3, and *p53* and significant reduction of the anti-apoptotic *Bcl2* gene with best apoptotic effect for the aqueous extract. Extracts used in the present study increased *Bax*, which induced the release of mitochondrial cytochrome c into the cytoplasm, further activating caspase3 expression [51]. Thus, these extracts could inhibit MCF7 proliferation via the initiation of the intrinsic pathway of apoptosis. However, the extrinsic apoptotic pathway cannot be overlooked. Therefore, further investigations on caspase 8 and Fas-L are needed. These results are in agreement with previous reports on the potent anticancer effect of *M. domestica* larvae extract and hemolymph on CT26 and MCF7 cancer cells [11,15] and *C. albiceps* larval extracts on a large variety of cancer cells including MCF7 [52].

Disrupting redox homeostasis is a central phenotype of several pathological states. Excessive oxidation harms different cellular components and is considered the hallmark for many diseases [53]. Reactive oxygen species (ROS) induce oxidative stress, which degrades proteins, lipids, and DNA [22,54,55]. The body prevents the over-release of free radicals and subsequently restricts oxidative stress through the activation of endogenous antioxidant enzymes (such as CAT, SOD, and GPx). Insects and their developmental stages (larvae and pupae) possess a powerful endogenous antioxidant system with a predominant effect for SOD [56,57]. Consistent with these findings, we also found that *V. orientalis* larval extracts possessed potent total antioxidant properties, as revealed by the DPPH assay. This effect was further proved on enzymatic and mRNA levels, and the results showed significant elevation in activities of antioxidant enzymes and expression of *NrF2* and *HO-1* genes following treatment with larval extracts. We also demonstrated a significant reduction in intracellular ROS as an indicator for oxidative stress. Similarly, Dutta et al. [32] found potent antioxidant properties for *V. affinis* aqueous pupal extract with higher SOD, CAT, and GST activities in human plasma (in vitro) and lower intracellular ROS in monocytes. El-Garawani et al. [11] also reported an antioxidant effect for *M. domestica* larval hemolymph with higher levels of SOD, TAC, and GSH and lower lipoperoxidation marker MDA levels. Several other studies also showed antioxidant effects for insect larval extracts such as blowflies [58] and beetles [59]. Interestingly, the increased anticancer potential of the aqueous extract was associated with a higher antioxidant status, which could be due to the existence of the defense constituents in larvae. In support, antioxidants can hinder tumorigenesis and growth and trigger apoptosis in cancer cells [11,23,60]. In general, the inhibitory effect on excessive ROS release and induction of antioxidant enzymes imply an advantageous role of *V. orientalis* larval extracts against oxidative stress-dependent diseases and cancer.

One of the main features of a potent anticancer drug is its ability to not only kill cancer cells, but also to prevent metastasis. Most mortality from cancer may be related to metastasis and the subsequent multiorgan dysfunction. Interestingly, *V. orientalis* larval extracts inhibited MCF7 metastasis as noticed by the results of both wound healing assay (which showed a humbled migration) and qPCR (reduced expression of the migration-related gene *MMP9* and increased expression of the anti-migration gene *TIMP1*). Unlike apoptotic and antioxidant effects, which revealed a leading effect for aqueous extract, no significant difference was noticed between the two extracts with respect to their anti-migration potential. Consistent with our results, cantharidin, a toxin extracted from beetles, also inhibited migration and invasion of human lung cancer cells through selective prevention of the expression of some MMP members [10].

The results obtained from qPCR revealed an anti-inflammatory effect for the larval extracts, as demonstrated by the downregulation of inflammation-related genes (*NFκB* and *IL8*), but with a predominant effect for the alcoholic extract. Using a similar mechanism, *V. orientalis* venom also possessed an anti-inflammatory effect [61]. Additionally, aqueous extracts of the house cricket, grasshopper, and silk moth contain notable anti-inflammatory and antioxidant activities [17,18]. Cancer cells induce inflammation via their ability to release inflammatory cytokines into the tumor microenvironment [62,63]. Inflammation plays an important role in cancer initiation and metastasis. When MCF7 is exposed to cytokines for long period, they undergo epithelial-mesenchymal transition, thereby facilitating cancer cell migration and metastasis. Hence, inhibition of cytokines such as *NFκB* hinders breast cancer cell migration [46,64]. Taken together, we provide *V. orientalis* larval extracts as novel inhibitors for chemotaxis-based migration of MCF7 cells.

HPLC analysis revealed the presence of several flavonoids and phenolic compounds, with the highest concentrations being for resveratrol and naringenin in the aqueous extract and rosmarinic acid in the alcoholic extract. In addition to other unknown higher peaks which worth further investigation. Thus, we suggest that the anticancer and antioxidant properties of these extracts could be attributed, at least in part, to these flavonoids and phenolic compounds. High levels of resveratrol and naringenin in the aqueous extract could explain its predominant anticancer and antioxidant potential relative to the alcoholic extract. Resveratrol has an anticancer effect and can potentiate the chemotherapeutic potential, reduce multidrug resistance, and limit metastasis when used in combination with standard anticancer drugs (reviewed in [65]). Resveratrol also has potent antioxidant properties that are mediated via activation of Nrf2 and its downstream target *HO-1* in rat PC12 cells and normal human breast epithelial MCF10F cells [66]. It also induces the p53-dependent pathway in cancer cells [67]. Similarly, we also found that the resveratrol-rich aqueous extract inhibited MCF7 proliferation, and this effect was accompanied by higher expression of *Nrf2*, *HO-1*, and *p53*. Naringenin inhibits the viability of a large variety of cancer cells and diminishes their migration and invasion [68]. It has been considered to be a perfect surgical adjuvant treatment for breast cancer patients, as it can modulate patient immunity and subsequently preventing tumor metastases after surgery [69]. Its anti-metastasis effect was associated with AKT pathway inhibition and downregulation of MMP2 and MMP9 [68]. On the other hand, the abundant amount of rosmarinic acid in the alcoholic extract could justify its superior anti-inflammatory effect. Rosmarinic acid possesses anti-inflammatory and anticancer properties against several cancer cell lines [70]. It can also cease the migration of MDA-MB-231BO human breast cancer cells from the breast to the bone through inhibition of *NF*κ*B* ligand (RANKL), osteoprotegerin, and IL8 expression [71]. Due to its potent anti-inflammatory effect, rosmarinic acid has been used in the treatment of inflammatory diseases such as arthritis, colitis, rhinitis, and acute pancreatitis through inhibition of cytokines including *NF*κ*B*, *IL1b*, and *IL8* [72]. In parallel, we also reported that the rosmarinic acid enriched alcoholic extract minimized MCF7 growth, and this effect was associated with the downregulation of *NF*κ*B*, and *IL8*.

This study provides new details regarding the anticancer effect of *V. orientalis* larval extracts against only one type of breast cancer cells (MCF7). However, it is worth confirming whether this effect also exists on other breast cancer cell lines to show that the observed effects are not only related to certain features in MCF7. Similarly, the effect of *V. orientalis* larval extract needs to be verified on animal models of breast cancer. Therefore, further in vitro and in vivo investigations are required to provide more mechanistic details in that regard.

## 5. Conclusions

Cancer cells maintain their viability via inhibition of apoptosis, initiation of inflammation, and migration. Stimulating apoptosis and targeting inflammation and migration can inhibit the proliferation of cancer cells and limit tumor progression. To the best of our knowledge, this is the first study to report that treatment with different *V. orientalis* larva extracts could inhibit MCF7 proliferation through induction of apoptosis, activation of antioxidant status, and inhibition of migration and inflammation. The aqueous extract showed the most potent anticancer effect, with a higher antioxidant effect but lower anti-inflammatory properties than the alcoholic extract. Thus, *V. orientalis* larval extracts could be useful in the future as adjuvants for breast anticancer drugs and as antioxidant and anti-inflammatory agents in the treatment of oxidative stress and inflammation-related diseases. However, further in vivo preclinical studies are required to verify this effect in animal models and check whether these extracts could be clinically relevant and safe for patients.

## Figures and Tables

**Table 1:** Primer sequences used in qPCR.

Gene	Forward Primer (5′–3′)	Reverse Primer (5′–3′)
Bax	GGACGAACTGGACAGTAACATGG	GCAAAGTAGAAAAGGGCGACAAC
Bcl2	TTGATGGGATCGTTGCCTTATGC	CAGTCTACTTCCTCTGTGATGTTG
Cas3	GAAGCGAATCAATGGACTCTGG	GACCGAGATGTCATTCCAGTGC
p53	TAACAGTTCCTGCATGGGCGGC	AGGACAGGCACAAACACGCACC
NrF2	CAGCGACGGAAAGAGTATG	TGGGCAACCTGGGAGTAG
HO-1	CGGGCCAGCAACAAAGTG	AGTGTAAGGACCCATCGGAGAA
MMP9	GCCACTACTGTGCCTTTGAGTC	CCCTCAGAGAATCGCCAGTACT
TIMP1	GGGCTTCACCAAGACCTACA	TGCAGGGGATGGATAAACAG
NFκB	ATGGCTTCTATGAGGCTGAG	GTTGTTGTTGGTCTGGATGC
IL8	ACTGAGAGTGATTGAGAGTGGAC	AACCCTCTGCACCCAGTTTTC
GAPDH	GGTGAAGGTCGGAGTCAACG	TGAAGGGGTCATTGATGGCAAC

**Table 2:** Flavonoids and phenolic compounds composition of 5% aqueous and alcoholic larval extracts.

Compounds	Aqueous Larval Extract		Alcoholic Larval Extract	
	Retention Time (min)	Amount (mg/kg)	Retention Time (min)	Amount (mg/kg)
Catechol	5.40	-	5.40	-
p-Hydroxy benzoic acid	7.68	0.019	7.70	-
Catechin	8.92	0.011	8.88	0.873
Chlorogenic	9.30	-	9.30	-
Vanillic acid	9.88	0.114	9.70	0.679
Caffeic acid	10.15	0.020	9.93	0.675
Syringic acid	10.37	0.025	10.50	-
p-Coumaric acid	13.45	-	13.45	-
Benzoic acid	14.27	0.032	14.30	-
Ferulic acid	15.42	0.004	15.69	0.337
Rutin	16.79	0.375	16.70	-
Ellagic	16.90	-	17.08	0.920
o-Coumaric acid	17.43	0.029	17.40	-
Resveratrol	19.74	1.580	19.80	-
Cinnamic acid	20.20	-	20.20	-
Quercetin	21.20	0.350	21.60	-
Rosemarinic	21.81	0.679	21.83	34.031
Naringenin	22.56	0.819	22.40	-
Myricetin	23.58	0.396	23.48	-
Kampherol	24.70	-	24.70	-
Total		4.452		45.460

## Data Availability

Not applicable.

## Supplementary Materials

The following are available online. Figure S1: Expression of *Bax*, caspase3 (*Cas3*), *p53*, and *Bcl2* genes in Vero cells following treatment with 5% aqueous and alcoholic larval extracts as detected by qPCR. Data were normalized to the housekeeping gene (*GAPDH*) and expressed as the mean fold change ± SEM. Samples ran in triplicates in 3 independent experiments, *n* = 5.

## Sample Availability

Samples of the compounds are not available from the authors.
