# Supplementary material for: Oriental Hornet (Vespa orientalis) Larval Extracts Induce Antiproliferative, Antioxidant, Anti-Inflammatory, and Anti-Migratory Effects on MCF7 Cells
Source: Molecules. 2021 May 31;26(11):3303. doi: 10.3390/molecules26113303 (PMC8198668; doi:10.3390/molecules26113303)
Supplement: Supplementary file 1 [file molecules-26-03303-s001.zip › molecules-1215126-supplementary.pdf]

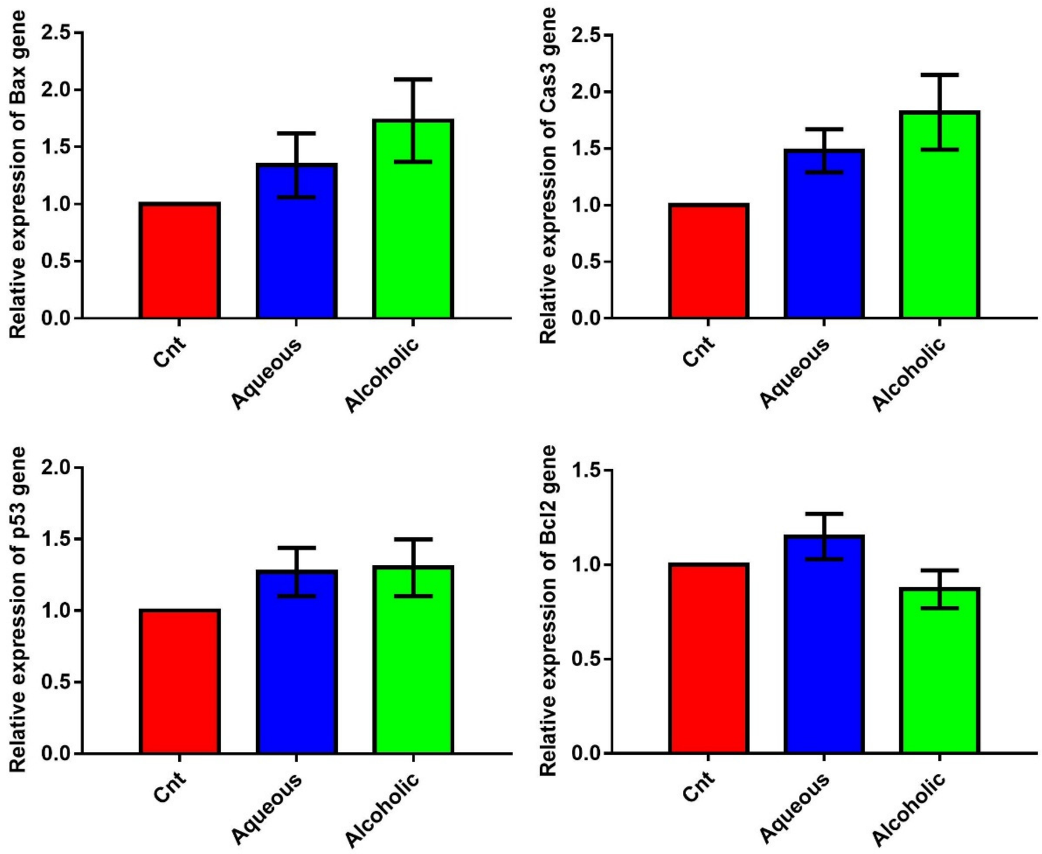

Figure S1. Expression of *Bax*, caspase3 (*Cas3*), *p53*, and *Bcl2* genes in Vero cells following treatment with 5% aqueous and alcoholic larval extracts as detected by qPCR. Data were normalized to the housekeeping gene (*GAPDH*) and expressed as the mean fold change ± SEM. Samples ran in triplicates in 3 independent experiments, n=5.
